# The sound of neural silence

**DOI:** 10.7554/eLife.111079

**Published:** 2026-04-30

**Authors:** Nikki Tjahjono, Yu-Shun Wang, Lin Tian

**Affiliations:** 1 Max Planck Florida Institute for Neuroscience Jupiter United States

**Keywords:** GABAergic transmission, fluorescent neurotransmitter sensor, extrasynaptic GABA, retinal direction selectivity, somatosensory cortex, high-throughput neuronal screening, Mouse

## Abstract

A new fluorescent sensor makes it possible to track the neurotransmitter GABA in freely moving animals.

**Related research article** Kolb I, Hasseman JP, Matsumoto A, Jensen TP, Kopach O, Arthur BJ, Zhang Y, Tsang A, Reep D, Tsegaye G, Zheng J, Patel RH, Looger LL, Marvin JS, Korff WL, Rusakov DA, Yonehara K, Team GP, Turner GC. 2026. iGABASnFR2 is an improved genetically encoded protein sensor of GABA. *eLife* **14**:RP108319. doi: 10.7554/eLife.108319.

A brilliant orchestra brings together many skilled musicians. Throughout a performance, each section must play at the right time and at the right volume to weave a unified melody. If everyone played whenever and however they wanted, the result would be cacophony.

The brain operates in a similar way. Billions of neurons communicate constantly, but not all signals occur simultaneously. To keep noise in check, an inhibitory neurotransmitter called GABA (short for gamma-aminobutyric acid) works alongside excitatory signals to maintain a vital balance between excitation and inhibition. When this balance is disrupted, the resulting ‘noise’ can lead to severe cognitive dysfunction (Wu and Sun, 2015). Therefore, to understand how the brain maintains this balance – and to understand how disturbing this balance can lead to disease – we need to learn more about how GABA is released.

Studying GABA requires tools that can precisely measure its concentration as a function of time and position in living animals. Traditionally, researchers have relied on microdialysis and patch-clamp electrophysiology for such measurements, but the former lacks temporal resolution and cellular specificity, while the latter lacks chemical specificity. To address this gap, a fluorescent sensor called iGABASnFR was developed (Marvin et al., 2019). The sensor combines a GABA-binding protein with a fluorescent protein: when GABA binds to the sensor, the fluorescent protein changes shape and becomes brighter. When expressed on neuronal membranes, the sensor detects changes in extracellular GABA concentrations. However, the original version had limited sensitivity and performance, especially for recordings in living animals. Now, in eLife, Glenn Turner (Janelia Research Campus) and colleagues – including Ilya Kolb and Jeremy Hasseman as joint first authors – report how they have improved the performance of this sensor (Kolb et al., 2026).

One version of the new sensor, called iGABASnFR2, increases fluorescence upon GABA binding, while the other becomes less fluorescent when GABA binds . Compared with the original sensor, iGABASnFR2 demonstrated improved membrane expression in neurons, faster response kinetics, greater affinity for GABA, and a wider dynamic range (i.e., it displayed larger fluorescence changes in response to GABA). These enhancements enabled successful imaging in both brain slices and living animals.

Kolb et al. then measured GABA signals at synaptic release sites, known as boutons, and demonstrated how inhibition shapes visual processing. Addressing a longstanding challenge in the field, they also successfully achieved live recordings of GABA release in the cortex of mice during sensory stimulation. The sensor is also compatible with fiber photometry, a technique that records neuronal activity in freely moving animals, opening new opportunities to link GABA signaling to behavior (Figure 1).

**Figure 1.**
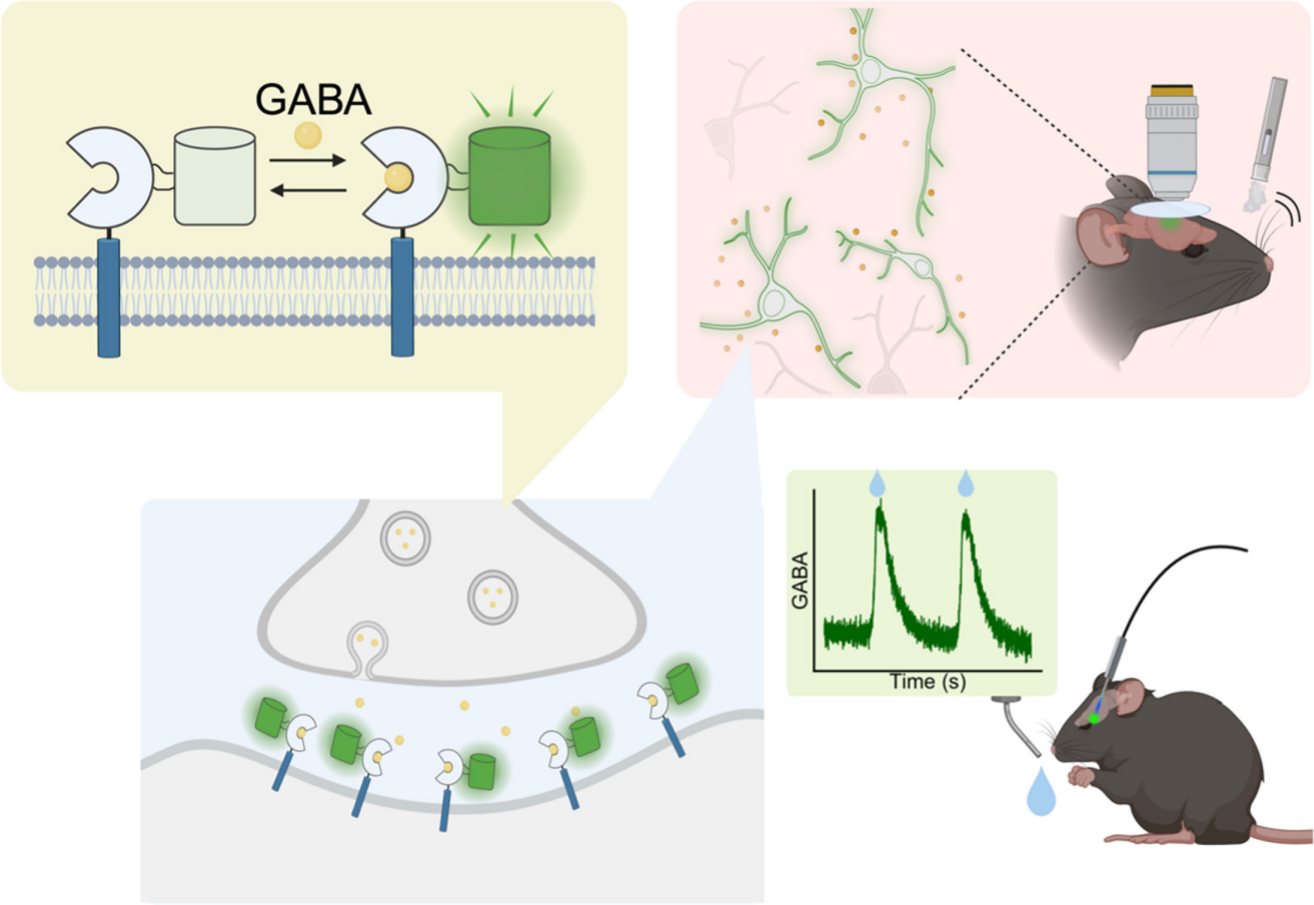
Next-generation sensors detect GABA across multiple scales. Top left: iGABASnFR2 is a membrane-bound sensor that contains a protein domain (the reversed C shape) that binds GABA (small circle) and a fluorescent protein (cylinder). When GABA binds to the first protein domain, the fluorescent protein becomes brighter. When iGABASnFR2 is expressed on a neuronal membrane, it can detect GABA molecules released by structures called boutons (bottom left). The fluorescence emitted by iGABASnFR2 can be imaged to track extracellular GABA in mice during various behavioural tasks (right). We thank Dr. Lesley Colgan (Max Planck Florida Institute for Neuroscience) for help with this figure.

Despite these advances, challenges remain. The sensitivity of iGABASnFR2 to GABA concentrations associated with tonic transmission could be improved. Unlike phasic transmission, which involves large changes in concentration on short timescales, tonic transmission occurs over longer time scales and involves small changes in ambient concentrations. The tonic transmission of GABA is thought to play a key role in regulating overall network excitability, but this process is not well understood (Glykys and Mody, 2007; Koh et al., 2023). Tonic transmission is particularly important in the cortex, where it helps establish the balance between excitation and inhibition that is critical for development and cognition (Antoine et al., 2019; Kim et al., 2021; Larsen et al., 2022). Tracking tonic GABA transmission in living animals will require biosensors that are both highly sensitive and stable over long periods.

Further improvements in GABA indicators will depend on a deeper understanding of its structure-function relationships. Kolb et al. resolved the crystal structure of iGABASnFR2, revealing how it binds to GABA. This structural insight can guide the design of new sensors. One promising direction is the development of lifetime-based sensors. Unlike intensity-based measurements, lifetime imaging does not depend on sensor concentration, enabling absolute, quantitative measurements of GABA levels rather than only relative changes (Ma et al., 2024). Future work may also produce red-shifted sensors operating at longer wavelengths, which would allow researchers to image GABA and other neurotransmitters and neuromodulators at the same time.

As efforts to map brain circuits advance, understanding inhibitory signals becomes increasingly important. Widely used technologies for recording brain activity can reveal where and when inhibitory neurons fire, but they do not detect when and where GABA is released. With sensors like iGABASnFR2, researchers can begin to identify the distinct roles of GABA, and tackle longstanding questions about how the excitation-inhibition balance is disrupted in conditions such as epilepsy, potentially guiding therapeutic development.
